# Prediction of Overall Disease Burden in (y)pN1 Breast Cancer Using Knowledge-Based Machine Learning Model

**DOI:** 10.3390/cancers16081494

**Published:** 2024-04-13

**Authors:** Seok-Joo Chun, Bum-Sup Jang, Hyeon Seok Choi, Ji Hyun Chang, Kyung Hwan Shin, Division for Breast Cancer, Korean Radiation Oncology Group

**Affiliations:** 1 Department of Radiation Oncology, Seoul National University Hospital, Seoul 03080, Republic of Korea; 2 Department of Radiation Oncology, Dongguk University Ilsan Hospital, Dongguk University College of Medicine, Goyang 10326, Republic of Korea; 3 Department of Radiation Oncology, Seoul National University College of Medicine, Seoul 03080, Republic of Korea; 4 Institute of Radiation Medicine, Seoul National University Medical Research Center, Seoul 03080, Republic of Korea

**Keywords:** Bayesian network, disease burden, disability weights, breast cancer, radiotherapy, de-escalation, in silico

## Abstract

**Simple Summary:**

We aimed to develop a Bayesian Network model to predict treatment outcomes and quality of life. Conditional probabilities and disability weights for radiotherapy-related benefit and risk were collected from nationwide expert survey. Overall disease burden (ODB) was defined as sum of conditional probabilities multiplied by disability weights. A Bayesian network model to predict ODB for (y)pN1 breast cancer was constructed. This model evaluated ongoing prospective trials for (y)pN1 breast cancer such as the Alliance A011202, PORT-N1, RAPCHEM, and RT-CHARM trials, validating reported results and assumptions.

**Abstract:**

Background: We aimed to construct an expert knowledge-based Bayesian network (BN) model for assessing the overall disease burden (ODB) in (y)pN1 breast cancer patients and compare ODB across arms of ongoing trials. Methods: Utilizing institutional data and expert surveys, we developed a BN model for (y)pN1 breast cancer. Expert-derived probabilities and disability weights for radiotherapy-related benefit (e.g., 7-year disease-free survival [DFS]) and toxicities were integrated into the model. ODB was defined as the sum of disability weights multiplied by probabilities. In silico predictions were conducted for Alliance A011202, PORT-N1, RAPCHEM, and RT-CHARM trials, comparing ODB, 7-year DFS, and side effects. Results: In the Alliance A011202 trial, 7-year DFS was 80.1% in both arms. Axillary lymph node dissection led to higher clinical lymphedema and ODB compared to sentinel lymph node biopsy with full regional nodal irradiation (RNI). In the PORT-N1 trial, the control arm (whole-breast irradiation [WBI] with RNI or post-mastectomy radiotherapy [PMRT]) had an ODB of 0.254, while the experimental arm (WBI alone or no PMRT) had an ODB of 0.255. In the RAPCHEM trial, the radiotherapy field did not impact the 7-year DFS in ypN1 patients. However, there was a mild ODB increase with a larger irradiation field. In the RT-CHARM trial, we identified factors associated with the major complication rate, which ranged from 18.3% to 22.1%. Conclusions: The expert knowledge-based BN model predicted ongoing trial outcomes, validating reported results and assumptions. In addition, the model demonstrated the ODB in different arms, with an emphasis on quality of life.

## 1. Introduction

The standard treatment for breast cancer is either breast-conserving surgery (BCS) followed by radiotherapy (RT) or mastectomy with or without RT [1,2]. The debate over adding regional nodal irradiation (RNI) for (y)pN1 disease persists due to variations in RNI extent across randomized trials and not all patients having pN1 disease. The rising use of neoadjuvant chemotherapy (NAC) [3] further complicates decisions, with a lack of randomized trials specifically addressing RNI addition for ypN1 disease. Controversies around pN1 disease in post-mastectomy radiotherapy (PMRT) echo those in BCS. While a meta-analysis by the Early Breast Cancer Trialists’ Collaborative Group suggested PMRT benefits in pN1 disease, these findings are based on trials from the 1980s, potentially differing from contemporary practices [4]. Hypofractionation poses concerns, particularly with limited information on hypofractionated PMRT amid an increasing trend of breast reconstruction after mastectomy [5,6].

Ongoing prospective trials aim to address radiotherapy-related issues in (y)pN1 breast cancer patients. In the Alliance A011202 trial (NCT01901094), patients with ypN1 breast cancer following positive sentinel lymph node biopsy (SLNBx) results are divided into groups undergoing axillary lymph node dissection (ALND) followed by chest wall (CW)/breast RT with RNI excluding the dissected axilla, and those undergoing full RNI with no further surgery. The PORT-N1 trial [7], a randomized phase 3 trial, randomizes pN1 disease patients who received either BCS or mastectomy to the control arm (received PMRT or whole-breast irradiation (WBI) with RNI), and the experimental arm (no PMRT or WBI alone). The prospective RAPCHEM trial [8] stratifies clinical N1 breast cancer patients into three risk groups based on pathologic nodal status after NAC. Different RT field strategies are employed for each risk group. The ongoing RT-CHARM trial (NCT03414970) aims to address the non-inferiority of reconstruction complication rates at 24 months between fractionation schemes of PMRT.

To predict outcomes of these ongoing trials, we employed an expert knowledge-based Bayesian network (BN) model, a probabilistic reasoning and machine learning model grounded in Bayes’ theorem. BN models, a type of machine learning algorithm, learn conditional dependencies between variables and estimate conditional probability distributions. This statistical framework enables predicting certain endpoints using conditional probabilities derived from prior studies or expert knowledge [9]. BN models also facilitate evaluating potential relationships among different nodes. Their application spans various fields, including diagnosis, risk assessment, and predictive modeling [10,11,12,13,14]. Combining prior knowledge with new evidence, BN models offer a robust framework for making predictions in uncertain clinical settings.

Given the ongoing nature of the trials described, their results will not be available for several years. Therefore, we utilized the BN model to predict outcomes, providing insights into patient risks and benefits in the absence of trial results.

## 2. Materials and Methods

### 2.1. Bayesian Network Model Design and Overall Disease Burden

The BN model developed for assessing the patients with (y)pN1 breast cancer comprised three key components: pretreatment, intervention, and RT risk/benefit. Patient- and tumor-related factors from the institutional data of (y)pN1 breast cancer patients diagnosed between 2020 and 2022 constituted the pretreatment component. The intervention involved categorizing the mitigation of the RT field into CW/breast alone, CW/breast + high-tangent, and CW/breast + internal mammary nodes (IMN)/supraclavicular lymph nodes (SCL). The RT-related risk and benefit component included disability weights and conditional probabilities for RT benefits (e.g., 7-year disease-free status) and costs (e.g., disease recurrence, reconstruction failure, and RT pneumonitis).

**Figure 1:**
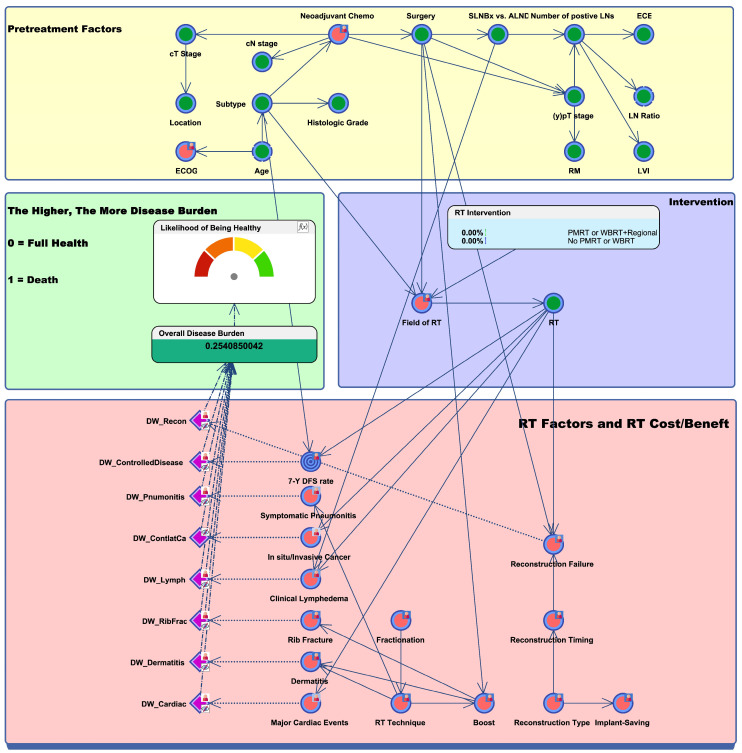
Bayesian network to represent radiotherapy-related risks versus benefits in breast cancer patients with (y)pN1 disease. The green nodes represent clinical factors collected by institutional data, while the red nodes represent factors collected by expert survey. The blue target nodes indicate the main endpoint of the study, which is 7-year DFS. Toxicity nodes are represented in purple. Key findings are summarized in the green box on the middle-left side, which demonstrates the likelihood of being healthy and overall disease burden. Abbreviations: Chemo, chemotherapy; SLNBx, sentinel lymph node biopsy; ALND, axillary lymph node dissection; LN, lymph node; ECE, extracapsular extension; RM, resection margin; LVI, lymphovascular invasion; ECOG, Eastern Cooperative Oncology Group; RT, radiation therapy; PMRT, post-mastectomy radiation therapy; WBRT, whole-breast radiation therapy; DW, disease weight; 7-Y DFS, 7-year disease-free survival.

Figure 1 provides an overview of the BN model tailored for patients with (y)pN1 breast cancer. Factors known to greatly influence other variables are connected by arrows. For instance, the field of RT was considered significantly influenced by the type of surgery (BCS or mastectomy) and molecular subtype (either triple-negative breast cancer [TNBC] or non-TNBC). Moreover, recognizing that RT can impact treatment outcomes and potential toxicities, we established connections between RT and other nodes, including 7-year disease-free survival (DFS), reconstruction failure, RT pneumonitis, and clinical lymphedema.

Disability weights for RT-related side effects and benefits were obtained through a nationwide expert survey, utilizing a scale from 0 to 1, where 1 represented the worst possible state. Experts were aided with examples from the 2019 Global Burden of Disease to determine disability weights. The values of the conditional probabilities and disability weights obtained from the survey were aggregated and integrated into the model. Within the program, probabilities and disability weights were then calculated using the provided confidence intervals. The overall disease burden (ODB) was defined as the sum of the product of disability weights and conditional probabilities for each RT-related risk and benefit. The likelihood of being healthy was calculated as 1 − ODB and linearly transformed to a probability from 0% to 100%. All conditional probabilities and disability weights are detailed in our previous work [15]. 

Model validation was omitted due to the lack of metrics, like sensitivity and specificity, dependent on data-learned parameters. Instead, rigorous review by three radiation oncologists was undertaken for each BN model component. The BN model was constructed using BayesiaLab 10.2 software (Bayesia S.A.S, Changé, France). 

### 2.2. In Silico Prediction of Trial Results

In a BN model, conditional probabilities can be estimated under various circumstances, allowing for the investigation of randomized trials and establishment of fixed probabilities between two interventions. We leveraged this property of the BN model to estimate oncologic outcomes, RT-related side effects, and ODB. The three randomized clinical trials included in the analysis were Alliance A011202 (NCT01901094), PORT-N1 [7], and RT-CHARM (NCT03414970). In addition to these randomized clinical trials, the RAPCHEM prospective cohort study is a previously published trial that provided 5-year follow-up data [8]. Because the study population of the RAPCHEM cohort overlaps with the current study, we compared these published results with those derived from the BN model.

## 3. Results

### 3.1. Outcome Inference of the Alliance A011202 Trial

**Figure 2:**
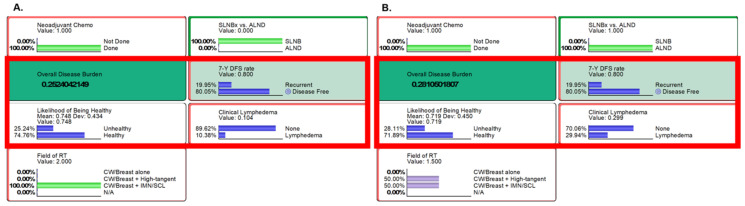
Results from the Alliance A011202 clinical trial settings. Inference using likelihood matching method are presented for (**A**) the first group [SLNBx followed by RT to CW/Breast plus IMN/SCL] and (**B**) the experimental group [ALND followed by RT to mixed state of RT to CW/Breast plus high tangent and IMN/SCL]. On the left (**A**): neoadjuvant chemotherapy was given, followed by SLNBx and RT+IMN/SCL. On the right (**B**): neoadjuvant chemotherapy was given, then ALND and RT (50% of high-tangent RT and 50% of RT+IMN/SCL) were followed. Key findings of overall disease burden, probabilities of likelihood of being healthy, 7-year DFS, and clinical lymphedema are compared, with an increase in overall disease burden observed in group B, mainly due to the increased risk of lymphedema. Abbreviations: Chemo, chemotherapy; SLNBx, sentinel lymph node biopsy; ALND, axillary node dissection; DFS, disease-free survival; CW, chest wall; IMN, internal mammary nodes; SCL, supraclavicular lymph nodes; N/A, not available; RT, radiotherapy.

The ongoing Alliance A011202 trial aims to assess non-inferiority between two treatments for ypN1 breast cancer post NAC. The first group underwent SLNBx followed by RT to CW/Breast plus IMN/SCL (Figure 2A), while the second group underwent ALND by RT to the mixed state of RT to CW/Breast plus high-tangent and IMN/SCL (Figure 2B). SLNBx group had a 7-year DFS of 80.1%, an ODB of 0.252, and a clinical lymphedema rate of 10.38%. ALND group resulted in a comparable 7-year DFS but an increased ODB (0.281) and 2.9-fold higher lymphedema probability.

### 3.2. Outcome Inference of the PORT-N1 Trial

**Figure 3:**
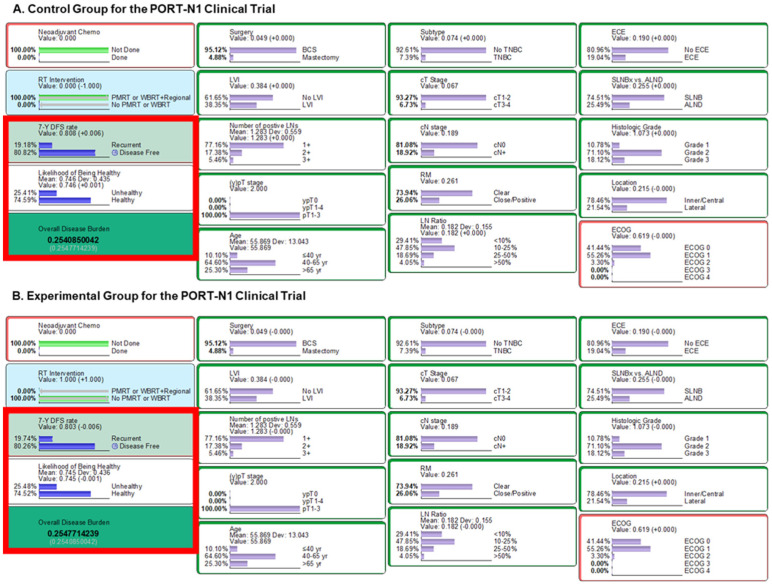
Results from the PORT-N1 clinical trial settings. Inference using likelihood matching method are presented for (**A**) the control group and (**B**) the experimental group. Abbreviations: LN, lymph node; Chemo, chemotherapy; WBRT, whole-breast radiation therapy; PMRT, post-mastectomy radiation therapy, TNBC, triple-negative breast cancer; BCS, breast-conserving surgery; 7-Y DFS, 7-year disease-free survival; SLNBx, sentinel lymph node biopsy; ALND, axillary lymph node dissection; LN, lymph node; ECE, extracapsular extension; RM, resection margin; LVI, lymphovascular invasion; ECOG, Eastern Cooperative Oncology Group.

The BN model maintained the probabilities of pretreatment factors, driving the randomization effect between both arms. We inferred that outcomes in the middle of NAC were “No” in accordance with the protocol of the PORT-N1 trial. We then observed an ODB of 0.254 in the control (PMRT or WBI+RNI) (Figure 3A) and 0.255 in the experimental group (no PMRT or WBI alone) (Figure 3B). In terms of oncologic outcomes, the 7-year DFS rates of the control and experimental groups were predicted to be 80.8% and 80.3%, respectively. 

We also performed a sensitivity analysis to compare the RT-related risk or benefit results for each arm of the PORT-N1 trial. The mean 7-year DFS rates were estimated to be 81.2% (Supplemental Figure S1A) and 80.6% (Supplemental Figure S1C) for the control and experimental arms, respectively. This trend was also observed in relation to the likelihood of being healthy. The mean likelihood of being healthy was predicted to be 74.6% for both groups (Supplemental Figure S1B,D). The predicted mean percentage of other side effects or risks following RT were similar between the two arms (Supplemental Table S1).

### 3.3. Outcome Comparison with the RAPCHEM Trial

We conducted a comparative analysis of ypN1 population outcomes obtained from the current BN model according to the policy outlined in the RAPCHEM study (Table 1). For patients with ypN1 disease who underwent ALND and received CW/breast RT, the 5-year locoregional recurrence rate was 2.2% according to the RAPCHEM study. The BN model estimated a 7-year DFS rate of 80.1%, along with an estimated ODB of 0.279 and a likelihood of being healthy of 72.1%. The RAPCHEM study did not provide specific information regarding patients who underwent SLNBx instead of ALND and received CW/breast RT. Nonetheless, the BN model estimated a lower ODB of 0.249 while maintaining a comparable 7-year DFS rate of 80.1%. Furthermore, when comparing the impact of RT on levels I/II and full regional nodes, the RAPCHEM study showed a 0.1% increase in the 5-year locoregional recurrence rate. Consistent with these findings, our BN model estimated a marginal decrease in the 7-year DFS rate from 80.3% to 79.0%. When comparing the effects of irradiation from level II to full RNI, our BN model revealed an increase in the ODB from 0.250 to 0.253 and a decrease in the likelihood of being healthy from 75.4% to 74.8%.

### 3.4. Outcome Inference of the RT-CHARM Trial

The RT-CHARM trial is evaluating non-inferiority of reconstruction complication rates at 24 months between conventional and hypofractionated PMRT. Because the BN model was not specifically designed for this situation, we were unable to detect any differences in the major reconstruction failure rate, as conventional and hypofractionated groups had a major failure rate of 20.9%. Table 2 summarizes 13 case scenarios that include RT-related factors such as boost, fractionation, contour for implant preservation, reconstruction timing, reconstruction type, and RT technique. Immediate reconstruction with RT had the highest major failure rate (22.1%), while delayed reconstruction after RT had the lowest (18.3%). 

## 4. Discussion

We developed an expert knowledge-based BN model to evaluate the ODB and risks/benefits of RT in patients with (y)pN1 breast cancer. We also performed in silico prediction of the outcomes of three ongoing randomized studies and one prospective registry study in terms of ODB, 7-year DFS, and RT-related side effects.

Several randomized trials, such as AMAROS and ACOSOG Z-0011, have explored the benefits of additional ALND in patients with node-positive breast cancer after SLNBx [16,17]. These trials have demonstrated no significant treatment advantage of ALND, suggesting that nodal disease burden can be adequately addressed through RT and systemic treatment post-SLNBx. As NAC is commonly administered [3], the role of ALND after NAC is being investigated in the Alliance A011202 trial. The current study found no difference in 7-year DFS between ALND and axillary RT in patients with pathologic node-positive disease after NAC. Notably, a substantial increase in lymphedema was observed in the ALND group, leading to a significant rise in the ODB. However, caution is advised in interpreting these findings, as retrospective studies have shown conflicting results when comparing additional ALND to SLNBx followed by RT [18,19]. Therefore, we await the results of ongoing trials to definitively address the de-escalation of ALND.

In patients with pN1 breast cancer, the study did not find a significant difference in the ODB when RT de-escalation was implemented in both mastectomy and BCS using the BN model. Although our study suggests that RT de-escalation does not significantly increase the ODB, it is important to consider the findings of multiple randomized trials and ongoing studies. Two notable trials, EORTC 22922 and Ma.20, have revealed treatment benefits of RNI [20,21]. Notably, not all patients included in these two landmark trials had pN1 disease, and the effectiveness of RNI was found to be lower in patients with pN+ disease than in those with pN0 disease. Thus, two ongoing trials, the Korean Radiation Oncology Group (KROG) 17-01 and the PORT-N1, are investigating RT de-escalation in patients with pN1 breast cancer [7,22]. The KROG 17-01 trial is a phase 3 trial that compares WBI alone to WBI with full RNI in BCS patients who received taxane-based chemotherapy. The PORT-N1 trial is another phase 3 trial that compares the control arm (PMRT or WBI with RNI) to the RT de-escalated arm (no PMRT or WBI alone) in both BCS and mastectomy patients. We anticipate that the forthcoming results of these trials will show no significant oncologic difference due to RT de-escalation, as to our BN model.

The RAPCHEM trial, focusing on RT de-escalation in cT1-2N1 breast cancer post NAC, demonstrated promising results, stratifying patients by locoregional risk [8]. Notably, 54% and 86% of intermediate- and high-risk patients, respectively, received RT according to the study guidelines [23]. Because most of the variation in outcomes was observed in patients with ypN1 disease, we specifically analyzed this patient population. We stratified patients into an intermediate-risk group (those with ypN1 disease after ALND) and a high-risk group (those with ypN1mi or ypN1 disease after SLNBx). According to our BN model, there were no significant differences in the 7-year DFS rate or ODB when the field of RT was varied. Interestingly, the BN model suggested that whole-breast/CW radiation with level I/II nodal irradiation may be a reasonable treatment option for patients with ypN1(mi) disease because it balances oncologic outcomes with the ODB.

The BN model additionally emphasizes the complex interplay between reconstruction factors and the likelihood of reconstruction failure after PMRT. Despite limitations in its original purpose, the model suggested reconstruction failure rates ranging from 18.3% to 22.1%, influenced by factors like the timing and type of reconstruction, as in previous studies [24,25]. The BN model found no significant difference between hypofractionation and conventional RT, consistent with a prior study [6]. This aligns with the ongoing RT-CHARM trial’s focus on evaluating reconstruction failure rates under different fractionation schemes.

The primary focus of ongoing trials is the de-escalation of ALND or RT in patients with (y)pN1 breast cancer. Both procedures can lead to an increased incidence of lymphedema, a substantial side effect identified by experts with a high disability weight [26,27]. In this study, the disability weight of lymphedema exceeded that of major cardiac events or reconstruction failure, emphasizing its impact on patient quality of life. The ODB tended to be elevated in scenarios involving ALND and RNI, as demonstrated by the model. Notably, lymphedema was expected to be present in almost 30% of cases involving ALND and RT, aligning with previous studies [26,27]. The model indicated no significant DFS advantage in the escalated RT or ALND arm, potentially favoring de-escalation due to its association with reduced lymphedema.

The BN model’s strength lies in its use of conditional probabilities, allowing the prediction of treatment outcomes and assessment of the ODB. The BN model, applicable across diverse clinical settings, leverages expert-based knowledge and design [10,11,12,13,14,28,29]. One notable advantage is its adaptability to new evidence, making it a valuable tool for interpreting outcomes of novel treatments or techniques. Indeed, another notable strength of the BN model is its ability to evaluate treatment outcomes for specific groups. This capability allows for stratification by selecting specific staging, hormone status, and other relevant factors. By doing so, we can assess both DFS and ODB, enabling a comprehensive estimation of treatment outcomes and related side effects. This level of granularity enhances the model’s utility in personalized medicine and clinical decision-making, as it provides tailored predictions for specific patient subgroups.

This study had three main limitations. First, certain simulated settings, such as axillary RT excluding the dissected ALND site, were not available because of the predefined divisions of the RT fields. Although we attempted to minimize possible discrepancies by manipulating the field of RT, variations may still exist. Second, the values used in the model were acquired through virtual simulations rather than direct observations. For example, minor factors known to impact toxicities were not connected in the nodes, indicating minimal influence on the outcome. Minor factors might have an impact on the precise prediction of the BN model. Lastly, the model could not be validated due to a lack of results from the ongoing studies. However, we have demonstrated that these simulated values align with findings from previous studies, adding credibility to the model predictions.

## 5. Conclusions

In conclusion, our in silico outcome predictions across several ongoing prospective trials consistently indicated trends of no significant differences in DFS and a decrease in ODB with treatment de-escalation. The in silico prediction of the Alliance A011202 trial revealed similar DFS rates but an increased incidence of lymphedema in patients undergoing ALND followed by axillary RT compared to SLNBx followed by axillary RT. Results from the PORT-N1 trial showed a 0.6% DFS benefit and no significant difference in ODB for patients undergoing BCS with RNI or mastectomy with PMRT compared to BCS with breast RT alone and mastectomy without PMRT. Additionally, the prediction of the RAPCHEM trial indicated similar findings to the actual results, advocating for breast/CW RT alone for intermediate- and high-risk patients. These findings support the non-inferiority of the de-escalated treatment for patients with (y)pN1 breast cancer. Our BN model predictions are also consistent with previously published results of prospective studies. Moreover, we showed distinct outcomes between treatment arms, highlighting the significance of patient quality of life in terms of the ODB.

## Figures and Tables

**Table 1:** Comparison between the results of the RAPCHEM trial and the Bayesian network (BN) model. Abbreviations: ALND, axillary lymph node dissection; CW, chest wall; DFS, disease-free survival; LRR, locoregional recurrence; N/A, not available; OBD, overall disease burden; RT, radiotherapy; SLNBx, sentinel lymph node biopsy.

	RAPCHEM Trial			BN Model		
Risk Group	Definition	RT	5-Year LRR	OBD	Likelihood of Being Healthy	7-Year DFS
Intermediate	ypN1 with ALND	Whole breast/CW	2.2%	0.279	72.1%	80.1%
		Whole breast/CW with level I/II	N/A	0.283	71.7%	80.3%
		Whole breast/CW with full regional RT	N/A	0.286	71.4%	79.9%
Intermediate or high	ypN1mi or ypN1 with SLNBx	Whole breast/CW	N/A	0.249	75.1%	80.1%
		Whole breast/CW with level I/II	2.2%	0.250	75.0%	80.3%
		Whole breast/CW with full regional RT	2.3%	0.253	74.8%	79.9%

**Table 2:** RT-related factors and the change in posterior probability of major reconstruction failure. Abbreviations: Recon, reconstruction; 3D-CRT, three-dimensional chemoradiotherapy; IMRT, intensity-modulated radiotherapy; RT, radiotherapy; Tomo, tomotherapy; VMAT, volumetric modulated arc therapy.

Boost	Fractionation	Implant Preservation	Recon Timing	Recon Type	RT Technique	Posterior Probability P (s|H)
No boost		Implant-preserving RT	Immediate			22.10%
No boost		No implant preservation	Immediate			22.10%
	Hypofractionated	Implant-preserving RT	Immediate			22.10%
			Immediate			22.10%
	Conventional			Autologous	IMRT/VMAT/Tomo	21.63%
No boost				Autologous	3D-CRT/field-in-field	21.63%
No boost				Autologous		21.63%
				Autologous		21.63%
No boost		No implant preservation				21.03%
		No implant preservation				21.03%
					IMRT/VMAT/Tomo	20.89%
No boost						20.89%
					3D-CRT/field-in-field	20.89%
	Conventional					20.89%
Boost						20.89%
		Implant-preserving RT				20.21%
				Implant-based		20.21%
			Delayed			18.29%

## Data Availability

The BN model has been uploaded to the Github repository (https://github.com/bigwiz83/BayesianNetwork_KROG22-13) (accessed on 26 September 2023).

## Supplementary Materials

The following supporting information can be downloaded at: https://www.mdpi.com/article/10.3390/cancers16081494/s1. Supplemental Table S1. Sensitivity analysis for the two arms of the PORT-N1 clinical trial; Supplemental Figure S1. Density plots for the probabilities between two arms of the PORT-N1 clinical trial. For the control group of the PORT-N1 trial, probability density of (A) 7-Year Disease Free Survival (DFS) and (B) likelihood of being healthy were plotted. For the experimental group, those of (C) 7-Y DFS and (D) likelihood of being healthy were plotted.
