# Supplementary material for: Prediction of Overall Disease Burden in (y)pN1 Breast Cancer Using Knowledge-Based Machine Learning Model
Source: Cancers (Basel). 2024 Apr 13;16(8):1494. doi: 10.3390/cancers16081494 (PMC11048634; doi:10.3390/cancers16081494)
Supplement: Supplementary file 1 [file cancers-16-01494-s001.zip › cancers-2928756-supplementary.pdf]

	Control group (PMRT or WBI + RNI)		Experimental group (no PMRT or WBI alone)	
	Mean	SD	Mean	SD
**7-year DFS rate**				
Recurrent	18.80%	23.90%	19.40%	22.80%
Disease-free	81.20%	23.90%	80.60%	22.80%
**Likelihood of being healthy**				
Unhealthy	25.40%	10.00%	25.40%	9.50%
Healthy	74.60%	10.00%	74.60%	9.50%
**Clinical lymphedema**				
None	84.60%	6.90%	84.90%	6.60%
Lymphedema	15.40%	6.90%	15.10%	6.60%
**Dermatitis**				
None or grade 1	80.70%	14.20%	80.70%	14.20%
Grade ≥ 2	19.30%	14.20%	19.30%	14.20%
***In situ*/invasive cancer**				
None	93.30%	5.40%	93.40%	5.20%
Contralateral event	6.70%	5.40%	6.60%	5.20%
**Major cardiac events**				
None	92.40%	7.20%	92.60%	6.80%
Major cardiac event	7.60%	7.20%	7.40%	6.80%
**Reconstruction failure**				
None	99.00%	0.60%	99.40%	0.40%
Major failure event	1.00%	0.60%	0.60%	0.40%
**Rib fracture**				
None	90.60%	11.70%	90.60%	11.70%
Clinical/radiological fracture	9.40%	11.70%	9.40%	11.70%
**Symptomatic pneumonitis**				
None	90.40%	8.70%	90.40%	8.70%
Symptomatic pneumonitis	9.60%	8.70%	9.60%	8.70%

Supplementary Table S1. Sensitivity analysis for the two arms of the PORT-N1 clinical trial. Abbreviations: DFS, disease-free survival; PMRT, post-mastectomy radiotherapy; RNI, regional nodal irradiation; SD, standard deviation; WBI, whole-breast irradiation.

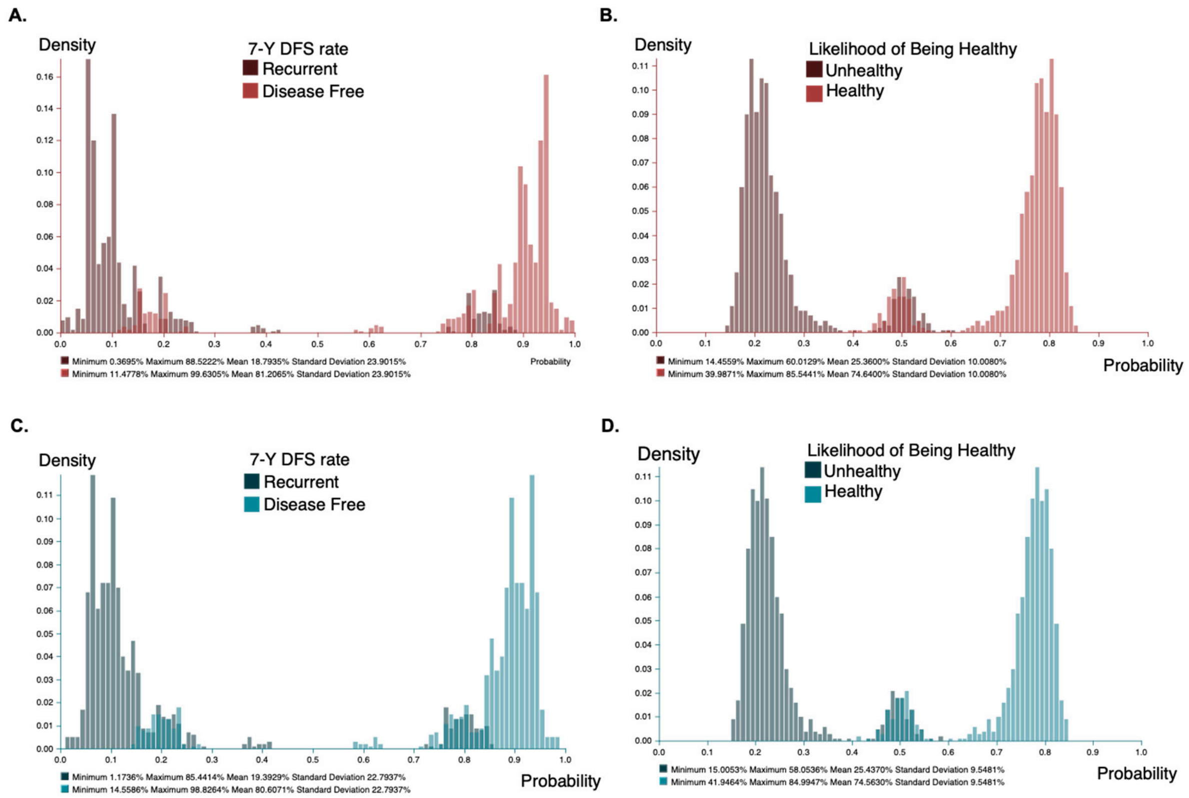

Supplementary Figure S1. Density Plots for the Probabilities between Two Arms of the PORT-N1 Clinical Trial. For the control group of the PORT-N1 trial, probability density of **(A)** 7-Year Disease Free Survival (DFS) and **(B)** likelihood of being healthy are plotted. For the experimental group, those of **(C)** 7-Y DFS and **(D)** likelihood of being healthy are plotted.
